# Perioperative neurocognitive dysfunction: thinking from the gut?

**DOI:** 10.18632/aging.103738

**Published:** 2020-08-15

**Authors:** Xiaolin Xu, Yimin Hu, Enshi Yan, Gaofeng Zhan, Cunming Liu, Chun Yang

**Affiliations:** 1 Department of Anesthesiology and Perioperative Medicine, The First Affiliated Hospital of Nanjing Medical University, Nanjing 210029, China; 2 Department of Anesthesiology, The Second Affiliated Changzhou People’s Hospital of Nanjing Medical University, Nanjing 213003, China; 3 Department of Anesthesiology, Affiliated Stomatological Hospital, Nanjing Medical University, Nanjing 210029, China; 4 Department of Anesthesiology, Tongji Hospital, Tongji Medical College, Huazhong University of Science and Technology, Wuhan 430030, China

**Keywords:** perioperative neurocognitive dysfunction, postoperative delirium, postoperative cognitive dysfunction, gut microbiota, brain-gut axis

## Abstract

With the aging of the world population, and improvements in medical and health technologies, there are increasing numbers of elderly patients undergoing anaesthesia and surgery. Perioperative neurocognitive dysfunction has gradually attracted increasing attention from academics. Very recently, 6 well-known journals jointly recommended that the term perioperative neurocognitive dysfunction (defined according to the Diagnostic and Statistical Manual of Mental Disorders, fifth edition) should be adopted to improve the quality and consistency of academic communications. Perioperative neurocognitive dysfunction currently includes preoperatively diagnosed cognitive decline, postoperative delirium, delayed neurocognitive recovery, and postoperative cognitive dysfunction. Increasing evidence shows that the gut microbiota plays a pivotal role in neuropsychiatric diseases, and in central nervous system functions via the microbiota-gut-brain axis. We recently reported that abnormalities in the composition of the gut microbiota might underlie the mechanisms of postoperative cognitive dysfunction and postoperative delirium, suggesting a critical role for the gut microbiota in perioperative neurocognitive dysfunction. This article therefore reviewed recent findings on the linkage between the gut microbiota and the underlying mechanisms of perioperative neurocognitive dysfunction.

## INTRODUCTION

Perioperative neurocognitive dysfunction (PND) refers to alterations in cognitive function before and/or after surgery, which are clinically manifested by abnormalities of learning, memory, language, thinking, spirit, and emotions [1]. Postoperative delirium (POD) and postoperative cognitive dysfunction (POCD) are now included in PND. PND has a higher incidence in elderly patients, which causes longer hospitalization, higher costs, a higher social burden, and even mortality. Factors that induce PND are capable of exacerbating the pathological changes in several neurodegenerative diseases, such as Alzheimer’s disease [2]. Although the reported incidence of POD and POCD varies widely, depending on the definition, test methods, and time points of evaluation, the incidence of cognitive dysfunction was up to 26% at 1 week after non-cardiac surgery, decreasing to 10% after 3 months [3].

The human host is a natural habitat for symbiotic microorganisms, including bacteria, fungi, and viruses. The number of microorganisms in the gut is about 10^13^-10^14^, and the total number of genes is approximately 10 times that of somatic cells [4]. The gut microbiota is a complex microbial system that exists in the human gastrointestinal tract and differs between individuals. It is involved in the regulation of multiple metabolic pathways, signal transduction, and immune inflammatory axes in the host [5, 6]. These axes are physiologically connected to the intestines, liver, muscles and brain [7]. The composition and activity of the gut microbiota are closely related to the host from the time of birth, and are subject to complex interactions over time, depending on the genome, nutrition, and lifestyle of the host. Imbalance in the gut microbiota affects human biochemistry, genetic personality, and disease resistance.

The microbial profile of the human gut at 1 year old might predict cognitive functions (especially in terms of communicative behaviours) at the age of 2 years, and may correlate with developmental disorders characterized by cognitive or linguistic delays [8]. Bacteria are rapidly implanted in the gut and continue to evolve and mature in childhood and adolescence; the brain is also at a critical stage of development, with implications for physical and mental health. Disrupting the host-microbiota symbiosis during these periods increases the risk of neurodevelopmental disorders, and is more susceptible to environmental factors, such as antibiotic use, stress, poor diet, and infection. Although the body and brain have matured during middle and old age, chronic progressive inflammatory response, drug use, degradation of digestion and gastrointestinal motility, malabsorption of nutrients, and impaired immunity during ageing will gradually reduce the diversity and stability of the microbiota. At the same time, aging is accompanied by a decrease in brain weight and cognitive function. Senescence-related changes in brain morphology are often seen in a variety of cognitive disorders associated with ageing, such as Alzheimer’s disease [9].

Given the prominent role of the gut microbiota in cognitive dysfunction, this review systematically summarizes recent findings regarding the relationship between the gut microbiota and PND.

## CONCLUSIONS

Perioperative medicine is increasingly regarded as a multidisciplinary field, and the occurrence of PND is also multifactorial. On the premise of the pathogenesis of PND, surgeons, anesthesiologists and nursing physicians are also constantly changing the management model for elderly patients. The findings of the interconnection between gut microbiota and PND, in prevention or treatment, is an important breakthrough. Although how gut microbiota plays a role in regulating PND through the brain-gut axis and how PND-related mechanisms and factors affecting gut microbiota have not been determined, gut microbiota is a promising viewpoint based on the pivotal role of gut microbiota in other cognitive disorders. Moreover, faecal microbiota transplantation (FMT) has also aroused widespread concerns in recent years, and its indications, methods, efficacy, safety, and ethics are also continually being explored and improved. Mature FMT technology will also facilitate the clinical application for diseases treatment. The application of gut microbiota for the prevention and treatment of PND needs further exploration.

## MATERIALS AND METHODS

PubMed was searched up to February 10, 2020 using the following keywords string: ‘cognitive dysfunction OR cognitive disorder’ AND ‘brain-gut axis’; ‘perioperative neurocognitive dysfunction OR postoperative delirium OR postoperative cognitive dysfunction’ AND ‘gut microbiota OR probiotics’; ‘cognitive dysfunction OR cognitive disorder’ AND ‘gut microbiota OR probiotics’. Relevant references were also retrieved for further analysis. From the published literature, we identified the themes that form the outline of our review. We selected the articles for inclusion based on a combination of the strength of evidence and the publication time in recent 10 years. Criteria for selection of papers were mostly depended on the influences of the papers.

### Brain-gut axis

The brain-gut axis is a bidirectional communication system regulating the function of the brain and gut [10]. It is well recognized that the brain-gut axis consists of the central nervous system (CNS), the central endocrine system, the central immune system, and intestinal microbes. This includes the hypothalamic-pituitary-adrenal (HPA) axis, the sympathetic nervous system, the parasympathetic nervous system (the vagus nerve), and the enteric nervous system (ENS) of the autonomic nervous system. Signalling from the gut can regulate some regions in the brain, such as the insula (related to self-perception), limbic system (associated with emotional processing), prefrontal cortex (linked with morality), amygdala (connected to fear), hippocampus (related to memory), and anterior cingulate cortex (related to positivity) [11–13].

The normal intestinal mucosal barrier has mechanical, chemical, immune, and biological components, made up of the intestinal mucosal epithelium, intestinal mucus, the gut microbiota, secretory immunoglobulin, and gut-associated lymphoid tissue (GALT). Abnormalities in the gut micorbiota are related to the occurrence of some neuropsychiatric diseases, such as irritable bowel syndrome (IBS), autism, obsessive-compulsive disorder, and depression [14, 15]. The mechanisms between abnormal composition of gut micorbiota and the mental changes caused by these diseases involves the gut-brain axis.

### Neural signalling pathway

The ENS is a complicated autonomic neural network of sensory, motor, and intermediate neurons that can independently regulate the basic functions of the gastrointestinal tract (movement, mucus secretion, and blood circulation). Studies have shown that intestinal microorganisms can affect the excitability of the intestinal myenteric nerves. The excitability of the neurosensory neurons is reduced in the absence of the intestinal microbiota in mice, while hyperpolarization is increased in antibiotics-induced pseudo-germ mice after the normal intestinal microbiota is transplanted [16]. Changes in the composition of the gut microbiota can also alter mood and cognition via the vagus nerve. Transplantation of *Campylobacter jejuni* into the intestine caused anxiety-like behaviour in a mouse model [17], while transplantation of non-pathogenic bacteria, such as *Bifidobacterium longum* into the duodenum had an antidepressant effect [18], but required a complete vagus nerve. Vagus nerve resection has become an effective treatment for certain neuropsychiatric diseases and refractory digestive diseases.

### Immune response

The intestinal mucosal immune barrier consists of intestinal mucosal lymphoid tissue (including mesenteric lymph nodes, and liver Kupffer cells) and intestinal plasma cell secretory antibodies (slgA). In addition to forming the intestinal physical barrier, the intestinal mucosal epithelial layer and the lamina propria are the largest immune cell bank in the body. The immune cell population in the epithelial layer is mainly composed of CD8^+^ lymphocytes, while the lamina propria includes macrophages, plasma cells, antigen presenting cells (APC), and mast cells [19]. Interestingly, T cells and APC can be transported from GALT to other lymphatic sites, and can cross the blood-brain barrier (BBB) [20]. It is well recognized that changes in intestinal microbial composition can disrupt the well-maintained balance between the microbiota and the host’s innate mucosal immune system, leading to activation of toll-like receptors and changes in cytokines that may lead to abnormal behaviour and cognitive dysfunction [21–23]. Cytokines are produced in the gut, and there is growing evidence that cytokines can enter the brain through the weak part of the BBB. They then activate the HPA axis to release cortisol via interleukin (IL)-1 and IL-6 [24]. Interestingly, it has been found that exosomes released by intestinal epithelial cells regulate the homeostasis of the gut microbiota and the adaptive immune response of the gut [25]. Moreover, the exosomes have great potential in neurological repair of stroke and in improving cognitive function in Alzheimer’s disease as they can pass through the BBB [26, 27].

### Microbial endocrinology

The intestinal microbiota can synthesize and release a variety of substances, including hormones, proteins, and neurotransmitters. *Lactobacillus* and *Bifidobacterium* can produce gamma amino butyric acid (GABA); *Escherichia*, *Bacillus,* and *Yeast* have the potential to produce norepinephrine; *Rosary Bacteria*, *Streptococcus*, *Escherichia coli,* and *Enterococcus* can produce 5-hydroxytryptamine (5-HT); *Bacillus* can produce dopamine; and *Lactobacillus* can produce acetylcholine [28, 29]. A variety of peptides secreted by the gut microbiota are involved in the regulation of circadian rhythms, feeding behaviour, sexual behaviour, arousal, and anxiety [30]. Growth hormone releasing peptide is thought to regulate the response of the HPA to stress [31, 32]. Galanin may be involved in physiological processes such as learning and memory, anxiety behaviour, repair and protection of nerve damage [33, 34]. 5-HT participates in the regulation of intestinal movement and pain perception peripherally, and maintains mood and cognition [35]. Drugs that increase serotonergic neurotransmitters (tricyclic antidepressants (TCAs) and selective serotonin reuptake inhibitors (SSRIs)) have shown therapeutic effects on emotional and gastrointestinal disorders [36].

### Bacterial metabolic pathways

Short-chain fatty acids (SCFA) produced by microbial fermentation of dietary fibre in the colon, including butyrate, propionate, acetate, are essential metabolites of intestinal microbial activity, and are trophic factors for the intestinal mucosal and epithelial layers [37]. SCFA can be transferred from the intestinal mucosa to the systemic circulation. They can induce inflammation and immune responses via the G protein-coupled receptors (GPR41 and GPR43) on the surface of intestinal epithelial cells and immune cells [38, 39]. SCFA can activate the sympathetic nervous system by binding to the GPR41 receptors in the sympathetic ganglion neurons [40, 41]. SCFA can pass through the BBB, affecting neurotransmission and the production of neurotransmitters, and can induce abnormal behaviours [42–44]. High doses of propionate in rats induces neuroinflammatory responses and behavioural changes associated with neurodevelopmental disorders, such as autism symptoms [45]. The bacterial metabolite lipopolysaccharide (LPS) can directly affect the CNS by activating the Toll-like receptor 4 in microglia, cause a mass-production of inflammatory cytokines in the CNS. Indirectly, it can induce the release of inflammatory cytokines from the gastrointestinal tract [46, 47]. Systemic IgA and IgM responses to commensal bacterial LPS were found in the blood of patients with depression and chronic fatigue syndrome, suggesting that LPS plays a major role in the pathogenesis of these diseases [48, 49].

The connection between brain and gut involves multiple disciplines and fields. In order to diagnose and treat neuropsychiatric diseases more accurately, it is necessary to further explore and study the mechanisms of the brain-gut axis, so as to figure out effective therapeutic strategies.

### The gut microbiota and cognitive dysfunction

**Figure 1:**
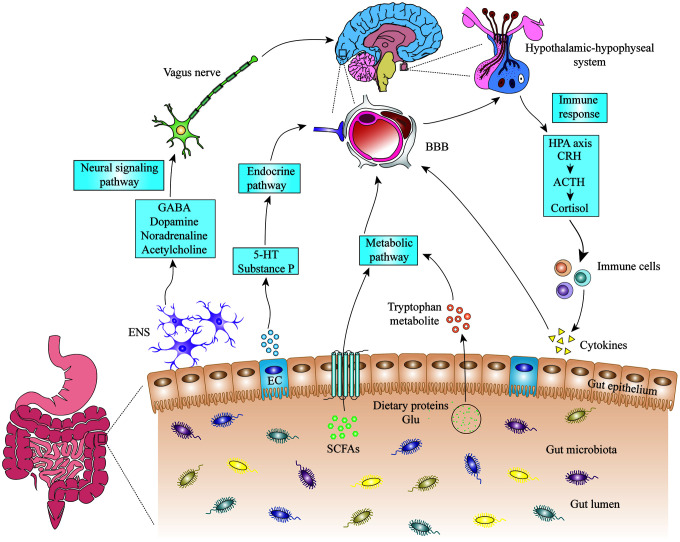
**The mechanisms of microbiota-gut-brain axis.** Gut microbiota can influence the cognitive function of brain through neural signalling, endocrine, metabolic and immune pathways. 5-HT: 5-hydroxytryptamine; ACTH: adrenocorticotropic hormone; BBB: blood-brain barrier; CRH: corticotropin releasing hormone; EC: enteroendocrine cell; ENS: enteric nervous system; GABA: gamma amino butyric acid; Glu: glutamic acid; HPA: hypothalamic-pituitary-adrenal; SCFA: short-chain fatty acids.

Cognition is the process of understanding and acquiring knowledge, involving a series of psychological and social behaviours such as learning, memory, language, thinking, energy, and emotion [50]. Cognitive dysfunction is the abnormality of these, accompanied by pathological processes such as aphasia, disuse, loss of recognition, and loss of behaviour. Common symptoms are hypersensitivity or dullness, over-memory or defect, association process disorder, logical thinking disorder, hallucinations, delusions, and a high risk of developing into dementia. The gut microbiota is involved in the development and progression of mental disorders that cause cognitive dysfunction [50, 51]. Although there may be differences in the results of intestinal microbiological studies due to factors such as region, diet, and individual patient differences, the effects of gut microbiota on cognitive function cannot be excluded (Figure 1).

### Gut microbiota and depression

Depression is a clinical type of affective disorder which is characterized by significant and persistent low mood, slow thinking, cognitive impairment, decreased willing and physical symptoms [52–54]. Although the cause of depression is not determined at present, scholars have explored the relationship between depression and gut microbiota. At present, a large number of studies on the intestinal microbial diversity of depression indicate that *Bacteroides*, *Proteus* and *Actinomycetes* are positively correlated with the occurrence of depression, while *Firmicutes* are negatively correlated with the occurrence of depression, and changes in group proportions of these bacteria can affect the occurrence and development of depression [55–59]. Humanized depressed mice, colonized with human depression-associated flora (characterized by Firmicutes, Actinomycetes, and Bacteroides), were found that the metabolism of carbohydrates and amino acids is clearly disordered, suggesting that the gut micobiota can interfere with host metabolism [56]. With the further study of the rapid and long-lasting antidepressant effect and mechanism of ketamine and its metabolites, it has been found that the gut microbiota has also undergone a corresponding change in the antidepressant process of ketamine and its metabolites [54, 55, 60, 61]. We previously reported that in the lipopolysaccharide-induced inflammatory depression mouse model, the phylum *Actinobacteria* and the class *Coriobacteriia* are significantly associated with the increased immobility time in forced swimming test (FST), and it is believed that these two microorganisms may be potential microbial markers for the antidepressant effects of ketamine [62]. In addition, the release of peripheral pro-inflammatory cytokines caused by endotoxin and gut microbiota disorders, such as C-reactive protein (CRP), IL-1, IL-6, tumor necrosis factor (TNF)-α, etc., can increase the permeability of BBB, leading to neuroinflammation, causing loss or activation of astrocytes, thereby triggering neuropsychiatric symptoms [63, 64]. Furthermore, the expression of BDNF mRNA in mice with chronic gastrointestinal inflammation is decreased, while after administration of *Bifidobacteria*, the depression-like behavior and hippocampal BDNF levels in mice can be normalized [65]. Pretreatment of rats with *Lactobacillus farciminis* can reduce the increased levels of adrenal and adrenocorticotropic hormones induced by stress and alleviate depression-like symptoms by affecting the HPA axis [66].

### Gut microbiota and Alzheimer's disease

AD is a progressive neurological degenerative disease characterized by accumulation of amyloid beta (Aβ) plaque, hyperphosphorylation of tau protein, neurofibrillary tangles knots and activation of inflammatory cells in brain tissue and along the blood vessels, and ultimately neuronal and other brain cell death [67, 68]. Clinically, AD is distinguished by comprehensive dementia such as memory impairment, aphasia, apraxia, agnosia, visual spatial impairment, executive dysfunction, and personality and behavioral changes [69, 70]. Although the etiology of AD has not yet been clarified, it is certain that changes in the composition of the gut microbiota affect the brain and behavior of patients with AD. Experts found that the gut microbiota in the feces of Aβ precursor protein (APP) transgenic mice changed significantly, while the Aβ amyloid deposition was found to be decreased sharply in the brain of pseudo-aseptic APP transgenic mice. Importantly, colonization of the gut microbiota of APP transgenic mice into the intestinal tract of sterile APP transgenic mice increased brain Aβ amyloid deposition. While microbial populations from wild-type mice were colonized into sterile APP transgenes, intestinal microbes do not increase brain Aβ amyloid levels, suggesting that gut microbes are involved in the development of Aβ amyloid pathology [71]. In most of intestinal microbial studies of AD, bacteria such as *Bacteroidete* have been found to be related to the occurrence of AD [72–75]. Accumulation of Aβ is one of the main pathological features of AD, and the production and clearance of Aβ in the CNS are in a state of dynamic equilibrium. A large number of bacteria and fungi in the gut, such as *Escherichia coli*, *Salmonella enterica*, *Salmonella typhimurium*, *Mycobacterium tuberculosis* and *Staphylococcus aureus*, can secrete functional extracellular amyloid, LPS and other related pro-inflammatory pathogenic signals [76, 77], which increase the CNS and systemic amyloid levels, breaking the homeostasis, leading to Aβ accumulation [78, 79] and may trigger host immune responses and neuroinflammation, increasing the risk of AD [80]; In addition, clearance of Aβ protein may be hampered by defects in triggering receptors enriched in the plasma membrane of microglia/myeloblast-2 cells (TREM2) [81]. Furthermore, antibiotic interference can alleviate neuroinflammation and Aβ plaque deposition in the mouse models of AD [82].

### Gut microbiota and Parkinson's syndrome

PD, also known as paralysis agitans, is a common progressive degeneration of the nervous system in the elderly. The pathological characteristics are the accumulation of misfolded protein alpha-synuclein (αSyn) in brain cells. The principal clinical non-motor symptoms include depression, anxiety, cognitive impairment, hallucinations, indifference, sleep disorders and other autonomic symptoms [83]. Gastrointestinal inflammation often occurs before the onset of motor symptoms in patients with PD [84]. In recent years, more and more attentions have been focused on the hypothesis that PD originates from the intestine and spreads to the brain through different pathophysiological pathways [85]. Recently, medical researchers have found new evidence that PD originates in the intestine and transmits signals into the brain, misfolded αSyn in the intestine can travel from the small intestine to the brain via vagus nerve [86]. Blockade of this transmission route may be the critical to prevent PD [87]. As early as 1971, it was known that gut microbes could metabolize levodopa to dopamine and m-tyramine in the gastrointestinal tract of patients with PD [88]. A study further found that the tyrosine decarboxylase that converts levodopa to dopamine is mainly present in *Enterococcus* and *Lactobacillus* [89–91], and the presence of these gut microbiota will lead to the increase of effective dose of levodopa medication for PD treatment [92]. A large number of studies have found that changes in levels of gut microbiota were associated with the severity of motor and non-motor symptoms in PD patients [93–95]. Mice over-expressing αSyn have shown that gut microbiota is essential for motor deficits, microglial activation and pathology of αSyn, and that gut microbes from PD patients can aggravate physical injury in mice over-expressing αSyn [96]. In recent years, insufficient production of hydrogen (H_2_) by gut microbiota has been taken into account to play a role in the pathogenesis of PD. H_2_ is a bioactive gas with anti-oxidation, anti-apoptosis, anti-inflammatory, cytoprotective and signal transduction properties. However, the fecal microorganisms of PD patients are deficient in H_2_-releasing bacteria, such as *Prevotella* and *Clostridium*, which reduce endogenous H_2_. Animal experiments have shown that H_2_ can attenuate the acute and chronic neurotoxic effects of 1-methyl-4-phenyl-1,2,3,6-tetrahydropyridine (MPTP) or 6-hydroxydopamine on dopaminergic neurons and neuron fibers in the substanto-striatum pathway in PD rat model. Clinically, it has also been confirmed that H_2_ bubbling water can improve the score of PD symptoms in hospitalized patients [97].

### Perioperative factors and treatment strategies of POD

POD refers to an acute mental disorder that occurs 1 week after surgery or before discharge and conforms to the Diagnostic and Statistical Manual of Mental Disorders, fifth edition (DSM-5) diagnostic criteria. The occurrence of POD has an obvious time-dependent characteristic, usually beginning in the recovery room after anaesthesia, and the symptoms are obvious 1 to 3 days after surgery, mostly in the elderly over 65 years. Its principal manifestations are disturbance of consciousness, behavioural disorders, inability to concentrate, abnormal perception, and sleep cycle disorders, which cannot be explained by pre-existing or developing dementia. It is clinically divided into agitation type, quiet type, and mixed type. According to the DSM-5 diagnostic criteria, 15% to 53% of elderly patients have delirium after surgery, of which 70% to 80% are located in intensive care. At present, the recognition rate of POD is low, but its harmfulness has caused extensive concerns in the medical community. The ‘cognitive care’ procedure of the Australian Commission on Safety and Quality in Health Care, the National Institute of Health and Clinical Excellence (NICE)’s clinical guidelines, and the American Society of Anesthesiologists (ASA)’s ‘Brain Health Plan’ are dedicated to the identification of high-risk patients, prevention strategies, and research areas to reduce delirium [98]. The following briefly describe the perioperative factors related to the occurrence of POD found in the current studies.

### Preoperative factors

Preoperative factors related to POD usually include patients’ preoperative medication, basic comorbidities, and basic education. There is abundant evidence that serum homocysteine (Hcy) levels are higher in the elderly and are associated with neurological diseases [99–101]. It has been reported that hyperhomocysteinemia increased susceptibility to PND, while preoperative supplementation with VitB_12_ and folic acid reversed this susceptibility [102]. In addition, a positive vagally mediated dose-dependent relationship between baseline cholinesterase activity and immune response in the plasma of delirium patients was noted, and lower preoperative plasma cholinesterase activity should be considered as a risk marker for POD in elderly patients [103]. As we all know, statins are commonly used to reduce blood lipids, thrombosis, inflammation, and for immune regulation, and have protective effects on the CNS. The usage of statins before surgery can reduce the incidence of POD after cardiac surgery [104]. It’s surprising but understandable that patients with higher education levels may have better tolerance to multiple perioperative disturbances, due to higher cognitive reserves and possible neuroplasticity, and had a lower incidence of POD and POCD [105, 106].

### Intraoperative factors

The general idea is that anesthesia method and anesthetics can impaire cognition. However, general anaesthesia, regional anaesthesia (RA), and local anaesthesia (LA) did not differ significantly in the incidence of POD in patients undergoing vascular surgery [107]. Drugs commonly used during surgery such as atropine, antihistamines, cortisol, benzodiazepines, propofol, and opioids have been shown to induce delirium [108]. Target-controlled infusion of propofol has been widely adopted in clinical anesthesia. Although intraoperative sedation depth has no significant effect on 1 year postoperative mortality and gait function recovery of patients, it can affect the incidence of POD, with lighter sedation associated with a lower incidence of POD [109]. Moreover, the use of electroencephalography (such as auditory evoked potential index and bispectral index) during surgery to guide anesthesia may reduce the incidence of POD [110]. It was formerly widely believed that the mean arterial pressure (MAP) below the lower limit of cerebral autoregulation during cardiopulmonary bypass (CPB) was related to numerous complications that occurred after cardiac surgery [111, 112]. However, Hori et al. demonstrated that the level and duration of MAP beyond the upper limit of cerebral autoregulation during CPB were significantly associated with the risk of POD, and advocated maintaining MAP within the range of cerebral autoregulation to reduce the risk of delirium [113]. In addition, clinical measures such as the use of heparin, hypothermia, and glucose-containing cardioplegic solution could cause stress-induced hyperglycemia, which is considered to be an important cause of perioperative inflammation, and closely associated with POD [114]. Regardless of the fact that brain-derived neurotrophic factor (BDNF) decreased with prolonged surgery time in both delirium and non-delirium patients, the former decreased by a larger percentage. Therefore, it is considered that the decrease of BDNF in plasma during surgery is a predisposing factor for POD in elderly patients [115]. In addition, blood transfusion as a common treatment for intraoperative anaemia is also suggested to be a protective factor for delirium. The consistent association between lower intraoperative haemoglobin levels and higher risk of delirium may be related to the oxygen supply, material synthesis, and metabolism of the brain [116].

### Postoperative factors

Postoperative nursing and drug-assisted treatment are often regarded as an indispensable step to accelerate the patient’s recovery by medical care personnel. Studies have suggested that delirium occurring in the postanaesthesia care unit (PACU) can increase the rate of POD by 4 times, so strengthening management during the PACU stay may reduce the risk of PND [117]. In addition, in elderly patients admitted to the ICU after non-cardiac surgery, prophylactic low-dose infusion of dexmedetomidine can significantly reduce the incidence of POD without significantly increasing the prevalence of bradycardia or hypotension [118]. Conventional postoperative care is beneficial for functional recovery, while postoperative individualized exercise training has been shown to be beneficial for reversing postoperative cognitive impairment in elderly patients during acute hospitalizations [119].

According to the European Society of Anaesthesiology’s evidence-based guidelines, POD is preventable. Therefore, the optimization of preoperative physiological condition is not only beneficial for the intraoperative period, but also accelerates postoperative recovery, including the recovery of the neuropsychiatric state. We should find the possible causes of POD before surgery and try to correct them before surgery. We also need to avoid applying drugs that have a significant effect on the mind after surgery and depriving patients of sleep. Sufficient analgesia is necessary, but we should closely monitor and avoid adverse effects. Reducing the incidence of delirium not only reduces healthcare-related costs, but also prevent undesirable sequelae.

### Pathogenesis of POCD and treatment strategies

POCD refers to the CNS complications after anaesthesia and surgery, which are mainly manifested as a decrease in cognitive ability, mental behaviour, social ability, and other aspects compared with the preoperative levels. This was first proposed by P.D. Bedford in 1955 [120]. Until 1998, J.T. Moller and other scientists designated it as postoperative cognitive dysfunction in an international multi-centre study involving 12 European and American Medical Centres [3]. According to the latest diagnostic criteria of DSM-5, POCD is defined as a mild or severe neurocognitive disorder occurring within 30 days to 12 months after surgery. It can occur in patients of all ages and has a higher prevalence in elderly patients over 65 years old, and a significantly higher disability rate. However, the pathogenesis of POCD is still unclear.

### The role of inflammatory immune response and oxidative stress

Surgery and anaesthesia are strong initiators of inflammation. Tissue damage and oxidative stress due to surgery and anaesthesia can induce the release of local or systemic pro-inflammatory factors and the activation of corresponding inflammatory signalling pathways. Pro-inflammatory cytokines can take advantage of the specific receptors and transporters on the surface of endothelial cells of the BBB and directly cross the BBB. Consequently, they can induce microglial activation and neuroinflammatory responses, and affect cognitive function. Microglia, especially macrophages in the CNS, are the main source of pro-inflammatory cytokines and reactive oxygen species (ROS) in the brain [121]. Preclinical trials have shown that the expression of cannabinoid 2 receptor (CB2R), high mobility group box-1 chromatin protein (HMGB1), S100β, and the activation of microglia are all related to POCD. The corresponding antagonists or agonists can prevent microglial activation and experimentally related cognitive deficits [122–124]. Antioxidants, including hydrogen-rich saline, elamipretide (SS-31), iron chelator (DFO), were found through NF-kappa B, mitochondria, p38 lightning mitogen-activated protein kinase (MAPK) signalling to achieve the release of reactive oxygen and pro-inflammatory cytokines, finally alleviating POCD symptos [125–128].

### The role of the neurotransmitter system

Inhibition of neuronal plasticity, neuronal damage, and the maladjustment of central neurotransmitter homeostasis are all closely related to the occurrence of POCD. BDNF signalling can be inhibited by numerous pro-inflammatory cytokines through activating P38MAPK and NF-κB, thereby reducing neural regeneration and neuronal plasticity [129]. CPB technology used in cardiac surgery reduces the mortality of myocardial infarction and heart failure, but complications, such as POCD and gastrointestinal tract injury, are common. Activation of the α7 nicotinic acetylcholine receptor (α7nAChR) can significantly reduce neuronal apoptosis, the expression of pro-inflammatory factors, and the number of CD4^+^ IL-17^+^ cells induced by CPB, thus alleviating intestinal injury and POCD symptoms. However, deficiency of α7nAChR significantly aggravates the pro-inflammatory response and POCD caused by CPB [130, 131]. Interestingly, pre-treatment with the acetylcholinesterase inhibitor (donepezil) could prevent spatial memory impairment in aged mice by alleviating the down-regulation of choline acetylase (CHAT) caused by isoflurane [132].

### Stress and endocrine disorder theory

Early life adversities, an important stressor, not only can cause chronic diseases and affective disorder in children [133–135], but also may be an independent risk factor for POCD in adulthood, for example maternal separation. On the one hand, maternal separation can promote the release of sevoflurane-induced hippocampal cytokine, activation of astrocytes and of the NF-κB signalling pathways in adult rats; On the other hand, it altered the DNA methylation status of exon 17 in the glucocorticoid receptor (GR) promoter region, and reduced GR expression [136]. Circadian disruption or metabolic endocrine stress may be an important mechanism of POCD in following patients. In a study of patients over 60 years of age undergoing major non-cardiac surgery, it was found that changes in the circadian rhythm of cortisol levels were significantly correlated with the occurrence of POCD [137]. In addition, anaesthesia and surgery can delay the secretion of melatonin at night, seriously interfere with the normal circadian rhythm of melatonin, and eventually disrupt the normal sleep cycle of patients [138]. Furthermore, melatonin can normalize the time shift of the clock gene mRNA peak, enhance the expression of clock gene mRNA, restore the circadian rhythm of resting activity and body temperature in elderly mice, and alleviate isoflurane-induced cognitive impairment [139].

### The role of anaesthesia and surgery

Anaesthetics that block NMDA receptors or enhance γ-aminobutyric acid type A receptors (GABA(A)Rs) have been shown to cause extensive apoptotic neurodegeneration and hippocampal synaptic dysfunction in the brain during synaptic development. Animal studies of agents used in anaesthesia can cause apoptosis in the developing brain [140]. Etomidate can continuously enhance the effect of the α5 subunit-containing GABA(A)Rs, damage the hippocampal memory and synaptic plasticity, and affect memory after anaesthesia. Inhibiting α5GABA(A)Rs can completely reverse the memory deficit after anaesthesia [141]. Most volatile inhaled anaesthetics can have neurotoxic effects on the CNS. Sevoflurane and isoflurane induce central nervous inflammation by enhancing the permeability of the BBB, and by damaging cerebral vascular endothelial cells [142]. Sevoflurane can increase the activation of caspase-3 (a marker of apoptosis) in the brain of mice and increase the expression levels of amyloid precuser protein (APP) and Aβ. Isoflurane induces cognitive impairment and aging-related hippocampal inflammation in aged mice by activating the NLRP3-caspase-1 pathway [143]. Preclinical studies have found that surgery under desflurane anaesthesia may actually reduce neuroinflammation and cognitive dysfunction [144]. The surgical methods are also a major factor leading to POCD. Hovens et al. used male Wistar rats to find that cognitive dysfunction caused by abdominal surgery is limited to the hippocampal brain region, while cognitive dysfunction caused by cardiac surgery involves changes in the broader cognitive domain, including the hippocampus, hypothalamus and prefrontal cortex, and increased markers of systemic inflammation, such as neutrophil gelatinase-associated apolipoprotein (NGAL) [129]. In addition, during cardiac surgery, extracorporeal circulation, temperature management, anaesthetic dose, tissue ischaemia-reperfusion, regulation of cerebral blood flow, and oxygen saturation may all cause neuroinflammation and cognitive impairment [145]. Intraoperative and postoperative long-term mechanical ventilation increase the expression of peripheral and hippocampal inflammatory cells, activation of the apoptotic cascade, and reactive hyperplasia of the microglia by activating the vagus nerve and triggering type 2 dopamine receptors, which further aggravates cognitive decline [146, 147].

Current treatment strategies for POCD are early diagnosis and timely treatment. Preventive measures should be taken and adequate evaluation should be undertaken before surgery [148]. Intraoperative optimization using various indicators, such as BIS index and cerebral oxygen saturation monitoring, should be employed [149]. It is necessary to create a good postoperative environment, ensure patients’ sleep quality, and pay attention to nutrition, water and electrolyte balance, strengthen psychological guidance and support, and reduce or stop taking high-risk drugs that are likely to cause POCD.

### The role of gut microbiota in PND

In recent decades, scientists and food and drug industry have conducted extensive research on probiotics and their interactions with humans. Probiotics can be used as prevention and treatment methods for disorders, such as inflammatory bowel disease, IBS and depression [150]. Nine preclinical studies reported the role of gut microbiota in PND (Table 1). We previously used hierarchical cluster analysis to analyse the behavioural results of mice that had undergone abdominal surgery, so that the mice were divided into sham group, POD group and non-POD group, and 16S ribosomal RNA gene sequencing was performed on their faeces. It was found that the α-diversity and β-diversity of gut microbiota were different between the POD and non-POD groups. In addition, there were significant differences in 20 species of bacteria at 6 different levels between the POD and non-POD groups, including *Tenericutes*, *Mollicutes*, *Bifidobacteriales*, and *Gammaproteobacteria*. Furthermore, we found that the pseudo-germ-free mice (induced by antibiotics) exhibited abnormal behaviors, and the gut microbiota of the non-POD group could improve the abnormal behaviors of pseudo-germ-free mice, while the gut microbiota of the POD group did not do so [151]. The results further support the hypothesis that the gut microbiota plays an important role in cognitive functions. Similarly, we also studied the role of the gut microbiota in POCD caused by surgery and anaesthesia, and found that the types and quantities of gut microbiota in the POCD group were significantly less than that in the non-POCD group in elderly mice. Bacterial abnormalities may be involved in the pathogenesis of POCD [152]. In order to further confirm the role of intestinal microorganisms in POCD, mice pre-treated with compound antibiotics or mixed probiotics (VSL # 3) suggested that the changes of 8 bacteria caused by anaesthesia and surgery were restored, and this prevented learning and memory dysfunction after anesthesia and surgery [153].

**Table 1:** Gut microbiota and probiotics associated with PND.

**Model/Disease**	**Animal**	**Surgery & Anesthesia**	**Behavioral tests**	**Microbiota in PND**	**Probiotics**
**POCD (Yang et al., 2018)^[124]^**	SD rats (8 months old)	Abdominal surgery & 2% isoflurane and oxygen	NORT	**Phylum:** *Actinobacteria*↓	none
**POCD (Zhan et al., 2019)^[152]^**	C57BL/6J mice (18 months old)	Tibial fracture fixation & 2% isoflurane and 100% oxygen	OFT; MWMT	**Phylum:** *Tenericutes*↓**;** *Chlamydiae, TM7*↑ Genus: *Anaeroplasma, Dehalobacterium, Sutterella↓*; *Chlamydia*↑	none
**POD (Zhang et al., 2019)^[151]^**	C57BL/6J mice (8 weeks old)	Abdominal surgery & 1.4% isoflurane and 100% oxygen	OFT; EPMT; BFT	**Phylum:** *Tenericutes*↓ **Genus:** *Ruminiclostridium*, *Ruminococcaceae UCG 014*, *Desulfovibrio*↓;	none
**POCD (Meng et al., 2019)^[155]^**	F344xBN F1 rats (aged)	Laparotomy & 2.1% isoflurane	FCT; OFT	not mentioned	none
**POCD (Jiang et al., 2019)^[153]^**	C57BL/6J mice (18 months old)	Tibial fracture fixation & 2% isoflurane and 100% oxygen	MWMT;	*Eubacterium coprostanoligenes*, *Actinomyces*, *Bacteroides*, *Butyrivibrio*, *Parabacteroides*↑ *Alistipes*, *Ambiguous_axa*, *Lachnospiraceae_NK4A136*, *Lachnospiraceae_UCG*, *Anaeroplasma*↓	*Bifidobacterium breve, B. longum, B. infantis, Lactobacillus acidophilus, L. plantarum, L. paracasei, L. bulgaricus, Streptococcus thermophilus*
**POD (Liufu et al., 2020)^[157]^**	Mice (9 and 18 months old)	Laparotomy & 1.4% isoflurane and 100% oxygen	BFT; OFT; YMT; BMT	*lactobacillus*↓	*Lactobacillus rhamnosus GG*
**POCD (Yu et al., 2019)^[158]^**	SD rats (10 weeks old)	cardiac surgery & 3% pentobarbital sodium	OFT; MWMT;	*Saccharibacteria*, *Eubacteriaceae*, *Enterobacteriales*, *Escherichia/Shigella*, *Micrococcaceae*↑; *Lachnospiraceae*, *Paraprevotella*, *Oscillibacter*↓	*Bifidobacterium longum,* *Lactobacillus bulgaricus,* *Streptococcus thermophiles*
**POCD (Liang et al., 2018)^[154]^**	CD-1 mice (6 to 8 weeks old)	Laparotomy & 0.25% bupivacaine	BMT; FCT	not mentioned	none
**POCD (Fonken et al., 2018)^[156]^**	F344XBN F1 rats (3 and 24 months old)	Laparotomy & halothane	contextual fear conditioning pre-exposure paradigm	not mentioned	none

↑ indicates increase; ↓ indicates decrease.

Abbreviations. BFT: buried food test; BMT: Barnes maze test; EPMT: elevated plus maze test; FCT: fear conditioning test; MWMT: Morris water maze test; NORT: novel object recognition test; OFT: open field test; YMT: Y maze test.

At the same time, many scholars have also confirmed the effects of gut microbiota on cognitive function in other animal models. A study demonstrated that the prebiotic Galacto-Oligosaccharide mixture can effectively inhibit the increase of the levels of microglial markers M1 and M2 in the hippocampus induced by surgery. It can also increase the relative abundance of *Bifidobacterium*, *Actinobacteria*, *Lactobacillaceae*, and *Lachnospiraceae*, and decrease the relative abundance of *Ruminococcaceae*, thereby increasing the expression of BDNF, reducing neuroinflammation caused by surgery, and improving postoperative cognition [124]. There is increasing evidence that long-term use of antibiotics can lead to cognitive dysfunction by causing imbalances in intestinal flora. The antibiotic cefazolin is often used clinically 3 to 5 days before surgery to prevent perioperative infection. Based on this, a study explored the relationship between cefazolin and POCD, and found that cefazolin can alleviate systemic, brain and colon inflammatory reactions caused by laparotomy in mice, and potentially reduce postoperative memory and learning disabilities. But unexpectedly, cefazolin can impair learning and memory when used alone in mice without surgery, which may be related to transient gut dysbiosis [154].

**Figure 2:**
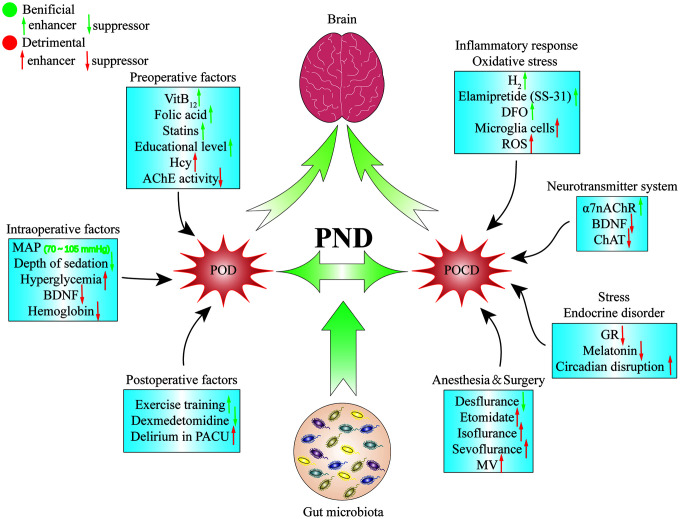
**The pathogenesis of perioperative neurocognitive dysfunction.** Postoperative delirium and postoperative cognitive dysfunction are two repensentive symptoms of perioperative neurocognitive dysfunction, and that multiple factors and pathways are probably involved in the pathogenesis of PND. α7 nAChR: α7 nicotinic acetylcholine receptor; AChE: acetylcholin esterase; BDNF: brain-derived neurotrophic factor; CHAT: choline acetylase; DFO: deferoxamine; GR: glucocorticoid receptor; Hcy: homocysteine; MAP: mean arterial pressure; MV: mechanical ventilation; PACU: postanaesthesia care unit; PND: perioperative neurocognitive dysfunction; POCD: postoperative cognitive dysfunction; POD: postoperative delirium; ROS: reactive oxygen species.

Trimethylamine N-oxide (TMAO) is a specific dietary nutrient metabolite derived from intestinal microorganisms. It is excreted by the kidney under normal physiological conditions. When the gut microbiota is imbalanced or renal function is impaired, TMAO levels in the circulation will increase, inducing oxidative stress and inflammatory response in the surrounding tissues. Recently, TMAO has been shown to further increase microglial-mediated neuroinflammation and hippocampal ROS production by reducing the expression of the antioxidant enzyme methionine sulfoxide reductase (Msr) A, leading to cognitive dysfunction in the elderly laparotomy group [155]. *Mycobacterium vaccae* *NCTC11659* (*M. vaccae*) is a saprophytic fungus that is found in the soil to regulate immune and anti-inflammatory effects. Interestingly, *Mycobacterium Vaccae* immunization not only plays a role in preventing depression-like behaviors caused by stress, but also enables the hippocampal microenvironment of old rats to change from a pro-inflammatory to an anti-inflammatory phenotype, reducing neuroinflammation and cognitive impairment caused by surgery [156] (Figure 2) [157, 158].
